# The therapeutic effect of bevacizumab on plaque neovascularization in a rabbit model of atherosclerosis during contrast-enhanced ultrasonography

**DOI:** 10.1038/srep30417

**Published:** 2016-07-25

**Authors:** Yang Li, Ying Zhu, Youbin Deng, Yani Liu, Yuhang Mao, Junli Wang, Jie Sun

**Affiliations:** 1 Department of Medical Ultrasound, Tongji Hospital, Tongji Medical College, Huazhong University of Science and Technology, Wuhan 430030, China

## Abstract

The purpose of the study was to assess the therapeutic effect of the angiogenesis inhibitor bevacizumab on plaques of various stages in rabbit models using contrast-enhanced ultrasonography (CEUS). Abdominal aortic atherosclerosis was induced in 55 rabbits. Thirty-six randomly selected rabbits were divided into 2 groups according to the timing of the bevacizumab injection: an early-stage plaque group (Group ESP) and a later-stage plaque group (Group LSP). The remainder were considered the control group. Standard ultrasonography and CEUS imaging of the abdominal aorta were performed. The animals were euthanized after CEUS, and plaque specimens were harvested for histological staining of CD31. The control group exhibited a substantially higher enhanced intensity, a higher ratio of enhanced intensity in the plaque to that in the lumen, and an increased number of CD31-positive microvessels in the plaque sections than Groups ESP and LSP (P < 0.05 for all). A higher enhanced intensity (P = 0.044), a higher ratio of enhanced intensity in the plaque to that in the lumen (P = 0.023) and more CD31-positive microvessels in the plaque sections (P = 0.006) were found in Group LSP than in Group ESP. Bevacizumab demonstrated more advanced inhibition of neovascularization in early-stage plaques in rabbits.

Clinical studies have revealed that a vulnerable plaque is closely associated with the incidence of acute cardiocerebrovascular events^1^,^2^. Plaque neovascularization has been well established and confirmed in many studies as a consistent feature of vulnerable plaques^3^,^4^. Intraplaque neovessels are at increased risk for haemorrhage because of their immaturity, inducing plaque rupture and instability, which may result in rapid progression to symptomatic disease^5^. These findings have advanced the development of potential treatments with angiogenesis inhibitors to reduce neovascularization for plaque stability. Some studies have demonstrated that angiogenesis inhibitors can counter plaque neovascularization based on the histological analysis of plaque specimens in animal models^6^,^7^. However, plaque neovascularization has not been assessed in living animal models of atherosclerosis after treatment with angiogenesis inhibitors, which is important for future clinical practice due to the ability to monitor the therapeutic real-time effects of these treatments. Furthermore, the inhibitory effect of plaques of different stages on neovascularization is unclear, and the influence of different doses on neovascularization has not been fully elucidated.

Advances in contrast-enhanced ultrasonography (CEUS) have enabled the detection of neovascularization within atherosclerotic plaques *in vivo*^8^,^9^. Several studies have reported that the enhancement within an atherosclerosis plaque observed during CEUS can be attributed to plaque neovascularization^10^,^11^. Moreover, the enhanced CEUS intensity correlates well with the microvessel density of atherosclerotic plaques in rabbit models^12^,^13^. Bevacizumab, a specific angiogenesis inhibitor that targets vascular endothelial growth factor (VEGF), has also been reported to decrease VEGF levels in rabbit serum and rabbit tissue^14^,^15^,^16^. Therefore, we conducted the present study to assess the therapeutic effect of bevacizumab on plaques of different stages in rabbit models using CEUS.

## Results

### Blood pressure, heart rate and serum lipids

In both Groups LSP and ESP, there were no differences in systolic and diastolic blood pressure, heart rate, total serum cholesterol (TC), triglyceride (TG), or low-density lipoprotein cholesterol (LDL-C) level before and after bevacizumab administration (Table 1).

### Ultrasonography findings

All experimental rabbits developed multiple plaques in their abdominal aortas. The control group exhibited a higher intimal-medial thickness (IMT) than Groups ESP and LSP (Table 2). Standard ultrasonography revealed 40 plaques in the control group, 43 plaques in Group ESP and 42 plaques in Group LSP. No significant difference in the maximum thickness of the plaques was observed among the three groups (Table 2). The control group exhibited a much higher enhanced intensity and ratio of enhanced intensity in the plaque to the abdominal aortic lumen compared with Groups ESP and LSP during CEUS. The enhanced plaque intensity and the ratio of enhanced intensity in the plaque to that in the lumen in Group LSP were significantly increased compared with Group ESP (Table 2). Furthermore, no changes were observed with respect to IMT (0.24 ± 0.05 mm vs. 0.26 ± 0.05 mm, P = 0.302), the enhanced intensity of the plaque (4.1 ± 1.53 dB vs. 4.9 ± 1.96 dB, P = 0.255) or the ratio of the enhanced intensity in the plaque to that in the abdominal aortic lumen (0.36 ± 0.15 vs. 0.45 ± 0.09, P = 0.169) in response to the different doses of bevacizumab.

### Morphometric evaluation

**Figure 1:**
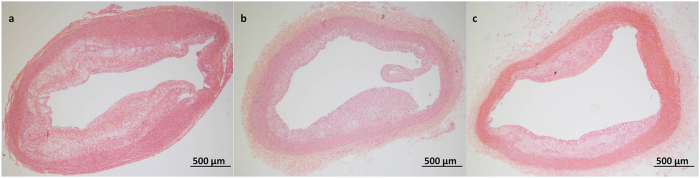
Representative micrograph of atherosclerotic plaque in the Control group (**a**), Group LSP (**b**) and ESP (**c**) (haematoxylin-eosin staining). No significant difference in plaque area, IMT or the maximum thickness of the plaques was observed between Groups ESP and LSP. The control group exhibited a higher IMT, maximum thickness of the plaques and a larger plaque area compared with Groups ESP and LSP.

No significant difference in plaque area, IMT or the maximum thickness of the plaques was observed between Groups ESP and LSP. The control group exhibited a higher IMT, maximum thickness of the plaques and a larger plaque area compared with Groups ESP and LSP (Fig. 1 and Table 3).

### Immunohistochemical analysis

**Figure 2:**
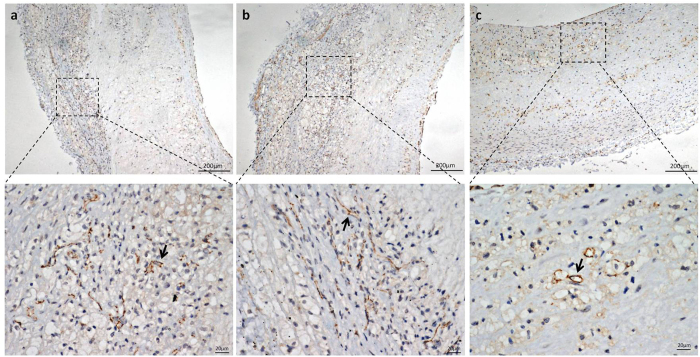
Representative immunohistochemical images of plaque in the Control group (**a**), Group LSP (**b**) and ESP (**c**) (CD-31 staining). Arrows in panel (a–c) indicated the intraplaque neovascularization. The total number of microvessels of plaques in the control group was greater than that in Groups ESP and LSP. Microvessels of the plaques in Group LSP were more than those in Group ESP.

**Figure 3:**
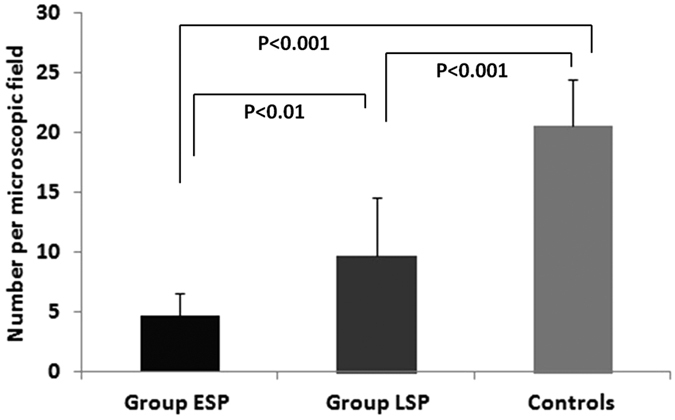
The quantification of CD31-positive microvessels in the plaque sections. The number of microvessels of plaques in the control group was greater than that in Groups ESP and LSP (p < 0.001). Microvessels of the plaques in Group LSP were more than those in Group ESP (p < 0.01).

The plaques in the control group contained a greater number of CD31-positive microvessels compared with those in Groups ESP and LSP. A greater number of CD31-positive microvessels were detected in Group LSP compared with Group ESP (Figs 2 and 3). No significant differences in neovascularization density were observed between groups receiving high-dose compared with low-dose bevacizumab (6.35 ± 4.18 vessels per field vs. 7.50 ± 4.14 vessels per field, P = 0.527). The enhanced intensity of atherosclerotic plaques and the ratio of enhanced intensity in the plaque to the lumen of the abdominal aorta were well correlated with the histological neovascularization density (r = 0.829, P < 0.001; r = 0.709, P < 0.001).

## Discussion

Many experimental *in vitro* and animal studies have suggested that angiogenesis inhibitors can suppress plaque neovascularization and atherosclerotic progression^6^,^17^,^18^; however, VEGF inhibition was recently reported to disrupt endothelial homeostasis and accelerate atherogenesis in animals^19^, and anti-VEGF therapy resulted in the acceleration of atherosclerosis in humans, as demonstrated by an increased IMT^20^. Therefore, at present the role of angiogenesis inhibitors in treating atherosclerosis is controversial. In the present study, no changes in blood pressure, heart rate or serum lipid were observed prior to compared with after bevacizumab administration. In comparison with the groups that received bevacizumab, the control group exhibited a much higher enhanced intensity, a higher ratio of enhanced intensity in the plaque to that in the lumen and a greater number of CD31-positive microvessels in plaque sections, suggesting that bevacizumab could inhibit neovascularization within atherosclerosis plaques without affecting blood pressure, heart rate or serum lipid levels. More importantly, our study demonstrated a more advanced inhibitory effect of bevacizumab on neovascularization in early-stage plaques and a dose-independent effect of bevacizumab on plaque neovascularization based on both the CEUS measurements and histological evidence.

VEGF is considered to be a potent regulator of neovascularization under both physiological and pathological conditions. Therefore, the inhibition of VEGF may eliminate plaque neovascularization and the consequent development of high-risk atherosclerotic plaques^16^. Bevacizumab is a humanized monoclonal antibody against VEGF-A ligand that binds to its circulating target, altering the kinetics of ligand binding to endothelial cells and downregulating angiogenesis^21^. Though it has been reported that therapies targeting VEGF were associated with hypertension, cardiotoxicity and thromboembolic events^22^,^23^; no side effects were observed in the groups that received bevacizumab during the experimental period. The blood pressure, heart rate and serum lipid levels of each rabbit did not change significantly after treatment with bevacizumab compared to baseline.

CEUS can be used to monitor real-time intraplaque neovascularization *in vivo* because of its noninvasive, high spatial and temporal resolution and the properties of the contrast agent microbubbles, which behave as pure intravascular tracers^1^. Moreover, the enhanced intensity correlates well with the microvessel density, with good intraclass correlations for the inter- and intra-observer agreement for enhanced intensity in rabbits^12^. Using CEUS, Pu *et al*.^24^ reported an inhibitory effect of anti-angiogenesis treatment on soft carotid plaque neovascularization in patients with non-small cell lung cancer, which was the first time CEUS was used to assess the inhibitory effect of angiogenesis inhibitors on plaque neovascularization. In that study, no histological evidence was provided to confirm the inhibitory effect of the anti-angiogenesis treatment. With the addition of present histological evidence, our work provides a more persuasive depiction of the inhibition of plaque neovascularization using CEUS in living animals.

The present results demonstrate a more advanced inhibitory effect of bevacizumab on neovascularization in early-stage compared with later-stage plaques. This finding highlights the vital role of early anti-angiogenesis therapy for the treatment of vulnerable plaques. Indeed, the diffusion of oxygen and other nutrients is limited to 100 μm from the lumen of the blood vessel, which, in vasa vasorum-derived microvessels, is adequate to nourish the inner media and intimal layers. As the plaque enlarges, the ensuing hypoxia and/or inflammatory cell infiltrate promote intraplaque neovascularization^25^, indicating more substantial neovascularization in later-stage compared with early-stage plaques. Therefore, the discrepancy in baseline microvessels contributed to our observed result that the inhibitory effect of bevacizumab on neovascularization was more effective in early-stage compared with later-stage plaques.

The present data suggest that there are no changes in intraplaque neovascularization in response to different doses of bevacizumab. This unexpected finding differed from a previous report showing that VEGF inhibits intraplaque microvessels in a dose-dependent manner^19^. This discrepancy may be associated with the similar dose of bevacizumab and the short-term observation period of our experimental protocol. Therefore, in future studies, a long-term observation period and distinct doses of bevacizumab will be key to elucidate the influence of different doses of bevacizumab on neovascularization.

Because the acoustic beam emitted from our probe could not completely penetrate the narrow intercostal spaces to provide valuable ultrasonoscopy, the cardiac function of rabbits was not assessed to identify any effects of bevacizumab on the heart. However, Pu *et al*.^24^ reported that the ejection fraction of patients with non-small cell lung cancer does not change following treatment with an angiogenesis inhibitor. We did not examine the difference in plaque neovascularization between the 5th and 9th weeks after balloon injury because at that time the plaque area was smaller and the assessment of plaque neovascularization during CEUS is subjective and associated with a risk of bias. Due to the lack of a natural comparison, a group of rabbits fed a normal diet and without aortic balloon endothelial denudation will be added in a future study. Additionally, because we could not evaluate the long-term efficacy of bevacizumab on plaques, longer observation periods will be necessary in future analyses.

This study demonstrates that bevacizumab could inhibit neovascularization within atherosclerotic plaques. A more advanced bevacizumab inhibition of neovascularization in early-stage compared with later-stage plaques was observed, highlighting the vital role of early anti-angiogenesis therapy in the treatment of vulnerable plaques. CEUS imaging may be used to monitor these phenomena in real-time *in vivo*.

## Methods

### Experimental protocol

The experiment protocol was carried out in accordance with the Animal Management Rule of the Ministry of Health, People’s Republic of China (documentation 55, 2001) and was approved by the Scientific Affairs Committee on Animal Research and Ethics of Tongji Hospital, Tongji Medical College, Huazhong University of Science and Technology (China). Abdominal aortic atherosclerosis was induced in 55 Japanese white rabbits (2.03 ± 0.11 kg, range 1.9–2.2 kg) provided by the Laboratory Animal Centre of Wuhan Research Institute of Biological Products through a combination of a cholesterol-rich diet and aortic balloon endothelial denudation.

On the 4th week after balloon injury, all of the experimental rabbits underwent standard ultrasound imaging of the aorta to confirm the success of animal models of atherosclerosis by detecting local thickening of IMT or small plaques. Then, thirty-six rabbits selected randomly were divided into 2 groups according to the timing of the bevacizumab injection: an early-stage plaque group (Group ESP, n = 18, in which bevacizumab treatment was applied to early-stage plaques), and a later-stage plaque group (Group LSP, n = 18, in which bevacizumab treatment was applied to later-stage plaques). The remaining rabbits were considered the control group (n = 19, without bevacizumab treatment). Group ESP was randomized into two subgroups based on the dose of bevacizumab: ESPh (n = 9, high-dose bevacizumab treatment), and ESPl (n = 9, low-dose bevacizumab treatment). Similarly, Group LSP was randomized into LSPh (n = 9, high-dose bevacizumab treatment) and LSPl (n = 9, low-dose bevacizumab treatment). On the 5th, 6th, 7th and 8th weeks after balloon injury, the rabbits in Group ESP were treated four times with a transvenous injection of bevacizumab (ESPh with 5 mg/kg, ESPl with 3 mg/kg^26^,^27^). Similarly, on the 9th, 10th, 11th, 12th weeks after balloon injury, rabbits in Group LSP were treated four times with a transvenous injection of bevacizumab (LSPh with 5 mg/kg, LSPl with 3 mg/kg). On the 14th week after balloon injury, all animals underwent standard and CEUS imaging of the aorta. The rabbits were euthanized after CEUS, and plaque specimens were harvested for immunohistochemical analysis.

### Establishment of aortic atherosclerosis

Balloon endothelial denudation was performed under ultrasonographic guidance as previously described^9^. All rabbits were initially fed a normal diet and deprived of food for 12h before the operation. Anaesthesia was induced and maintained by intravenous administration of 3% pentobarbital (1.5 mL/kg). The anaesthetic effect was monitored by observing the movement of the rabbits. Anaesthesia was maintained for 1–1.5 h based on the duration required for balloon endothelial denudation. The arterial blood pressure was monitored and recorded with a 21-gauge venous indwelling needle that was placed and fixed in the left femoral artery, which was connected to a pressure transducer connected to an electronic monitor (Dash4000, GE Healthcare, Milwaukee, WI, USA). The heart rate was monitored with a cardiotachometer that was triggered by arterial pressure waves. Experimental rabbits were fixed in the supine position. The right femoral artery was carefully dissected using an aseptic technique, and the balloon catheter was progressed via exposed femoral artery until the thoracic descending aorta was reached. The balloon was gently inflated with saline solution to maintain a pressure between 10 and 15 kPa and then slowly pulled back until iliac bifurcation was achieved and then forward until the thoracic descending aorta was reached. This was performed three times. Subsequently, the catheter was removed, and the right femoral artery was ligated. After balloon endothelial denudation, 80,000 units/d of penicillin were administered intramuscularly for 1 week. After revival, the animals were fed a cholesterol-rich diet (2% cholesterol, 3% egg yolk powder and 3% lard) for 14 weeks.

### Ultrasound imaging

Anaesthesia was induced by intravenous administration of 3% pentobarbital (1.5 mL/kg) during the ultrasound imaging. The arterial blood pressure and heart rate of each animal was monitored and recorded during both standard and CEUS imaging as described above. Standard ultrasonography and CEUS imaging of the abdominal aorta were performed as previously described^9^ on the 14th week after balloon injury with an ultrasound machine (Logiq E9, GE Healthcare, Milwaukee, WI, USA) using a 9L linear array transducer with a transmission frequency of 12 MHz for both standard and CEUS imaging. The ultrasound transducer was placed on the abdominal skin to visualize the right renal artery and the longitudinal plane of the abdominal aorta. To minimize image misalignment, the position of the ultrasound transducer relative to an anatomical landmark (right renal artery) was marked on the skin. If a plaque was identified, the view showing the thickest cross-section of the plaque was used to measure the maximum plaque thickness with electronic calipers. IMT was defined as the distance from the leading edge of the lumen-intima interface to that of the media-adventitia interface. The maximum plaque thickness was measured as maximal IMT, which was defined as the greatest axial thickness in the abdominal artery^1^. The distance between the plaque and right renal artery was measured and used for the identification of the selected plaques in repeated standard and CEUS imaging.

After standard ultrasound imaging, the animal underwent CEUS imaging of the previously identified plaques^9^. The preset real-time, contrast-enhanced imaging modality with coded pulse inversion technique (ver.3.1.2, GE Healthcare, Milwaukee, WI, USA) was switched on, and image settings were adjusted to maximize contrast signal visualization. To reduce microbubble destruction, we preset the mechanical index to 0.09 and the frame rate to 12/s. The image depth was adjusted to 1–2 cm according to the size of the abdominal aorta, and the focus position was set at the level of the abdominal aorta. All settings were kept constant throughout each study. SonoVue (Bracco, Geneva, Switzerland) was used as the contrast agent. The contrast agent was administered intravenously as a 0.4-mL bolus through the marginal ear vein, followed by a 1-mL bolus of saline. A real-time contrast-enhanced cine loop was acquired after injection of the contrast agent, including images obtained at least 3 s before and 120 s after the appearance of the contrast effect in the lumen of the abdominal aorta. The cine loop was digitally stored for later analysis.

### Imaging analysis

**Figure 4:**
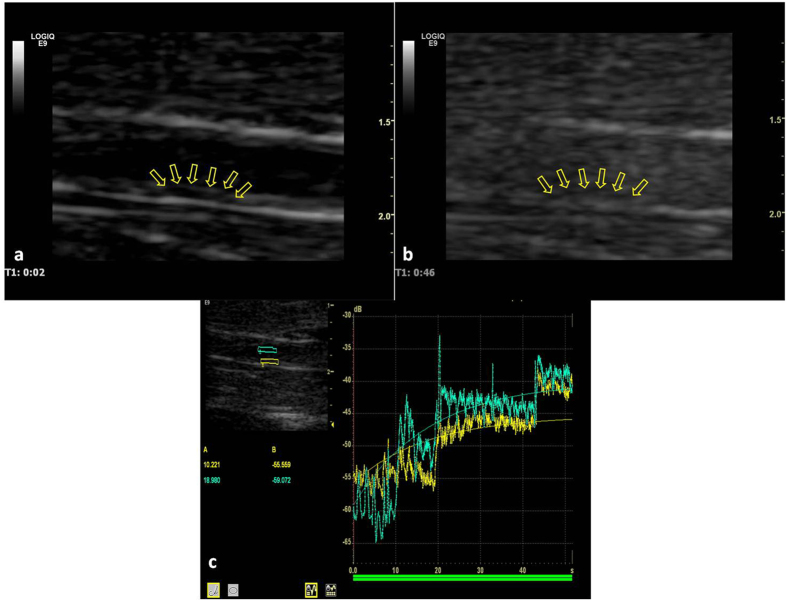
Contrast-enhanced ultrasound images of the abdominal aorta. (**a**) Longitudinal view obtained 2s after injection of contrast agent. No enhancement was observed in the plaque (arrows). (**b**) Longitudinal view obtained 46s after injection of contrast agent. Plaque (arrows) was enhanced after the injection of contrast agent. (**c**) The signal intensity-versus-time curve in the lumen of the abdominal aorta (blue area and curve) and plaque (yellow area and curve). A in panel c = enhanced intensity after injection of contrast agent; B in panel c = baseline intensity (background intensity) prior to the injection of contrast agent. When a plateau of the signal intensity-versus-time curve occurs, the derived peak signal intensity after injection of the contrast agent in the regions of interest and the baseline intensity (signal intensity at the origin of the curve) before injection of the contrast agent were obtained. The enhanced intensity was calculated by subtracting the baseline intensity from the derived peak signal intensity. The degree of enhancement of the plaque after injection of contrast material was investigated by measuring the enhanced intensity in the plaque and the ratio of the enhanced intensity in the plaque to that in the lumen of the abdominal aorta.

The enhancement of the plaque after injection of the contrast material was quantitatively analysed offline as previously described^1^ using a time–signal intensity curve analysis software package (ver. 7.0.5, GE Healthcare, Milwaukee, WI, USA) capable of displaying the signal intensity-versus-time curve in the region of interest during the enhancement process. The plaque outline was manually delineated as a region of interest (Fig. 4). A copy of the region of interest with the same size and shape as in the plaque was placed at the lumen of abdominal aorta near the plaque as a reference.

The abdominal aortic plaque and lumen signal intensity-versus-time curves during the process of enhancement were automatically produced and fitted to an exponential function: Y(t) = At·e^−kt^ + B, where Y is the signal intensity at time t, k is a factor proportional to the transit time of the contrast agent, A is the derived peak signal intensity, and B is the intercept signal intensity at the origin of the curve (baseline intensity or background intensity)^28^. The baseline intensity (background intensity) before injection of the contrast agent and the derived peak signal intensity after injection of the contrast agent in the regions of interest were obtained from the signal intensity-versus-time curve. The enhanced intensity was calculated by subtracting the baseline intensity from the derived peak signal intensity. The degree of enhancement of the plaque after injection of contrast material was investigated by measuring the enhanced intensity in the plaque and the ratio of the enhanced intensity in the plaque to that in the lumen of the abdominal aorta.

### Histological analysis of plaque specimens

Rabbits were sacrificed via an intravenous injection of an excessive amount of 3% pentobarbital (3.0 mL/kg) after ultrasound imaging.

After the abdominal aorta and right kidney artery were separated, previously identified plaques were harvested according to their distance from the right kidney artery measured using standard ultrasonography. All specimens were fixed in 2% paraformaldehyde for 24 h and embedded in paraffin. Subsequently, the tissue samples were serially sectioned (3 μm) and stained with haematoxylin-eosin for plaque morphometric evaluation and CD31 (1:50 dilution, Santa Cruz Biotechnology, Dallas, Texas, USA) to specifically identify neovascularization. The plaque images were analysed with the Image Pro plus system (ver. 6.0; Media Cybernetics, Silver Spring, MD, USA). Plaque area, IMT and maximum plaque thickness were evaluated. Plaque area was calculated as the difference between the luminal area and the area delimited by the internal elastic lamina. The results were normalized to the total vessel cross-sectional area for each arterial section to eliminate variations due solely to vessel size^18^. The neovascularization density on the digitized images was quantified by counting the total number of CD31-positive microvessels per 200× magnification microscopic field. Thirty plaques were randomly selected to analyse the linear relationship between the enhanced intensity and the histological intraplaque neovascularization density.

### Serum lipid level test

Rabbits were fasted over night before blood was drawn. Serum was separated by centrifugation and stored at −80 °C until analysis. TC, TG, and LDL-C levels were measured using an automatic biochemistry detection system before and after bevacizumab treatment.

### Statistical analysis

Data analysis was performed using SPSS software (ver. 15.0; SPSS, Chicago, IL). All values are expressed as the mean ± standard deviation. Parameters assessed in the ESP, LSP and control groups were compared by analysis of variance. Significant differences between groups were assessed using the Scheffe F test for multiple comparisons. Significant differences were compared between each group before and after treatment with bevacizumab using a paired t-test. An independent-sample t-test was applied to identify significant differences between groups that received low-dose versus high-dose bevacizumab. A Pearson correlation was used to assess the linear association between enhanced intensity and the histological intraplaque neovascularization density. Differences with a p value of less than 0.05 were considered significant.

## Additional Information

**How to cite this article**: Li, Y. *et al*. The therapeutic effect of bevacizumab on plaque neovascularization in a rabbit model of atherosclerosis during contrast-enhanced ultrasonography. *Sci. Rep*. **6**, 30417; doi: 10.1038/srep30417 (2016).

## Figures and Tables

**Table 1:** Blood pressure, heart rate and serum lipids before and after bevacizumab administration.

	Group ESP (n = 18)		Group LSP (n = 18)	
	before BV	after BV	before BV	after BV
SBP, mm Hg	84 ± 13	80 ± 13	82 ± 10	79 ± 11
DBP, mm Hg	56 ± 16	53 ± 17	55 ± 15	52 ± 16
HR, beats/min	260 ± 30	253 ± 18	255 ± 24	253 ± 19
TC, mmol/L	35.7 ± 1.9	36.9 ± 1.2	35.8 ± 1.7	36.3 ± 1.4
TG, mmol/L	4.1 ± 2.9	6.0 ± 3.6	4.0 ± 2.5	5.5 ± 2.7
LDL-C, mmol/L	36.6 ± 6.5	42.2 ± 1.6	36.3 ± 6.4	38.7 ± 6.3

Note: Data are expressed as the mean ± standard deviation. BV = bevacizumab; SBP = systolic blood pressure; DBP = diastolic blood pressure; HR = heart rate; TC = serum cholesterol; TG = triglycerides; LDL-C = low-density lipoprotein cholesterol.

There were no differences in blood pressure, heart rate, TC, TG or LDL-C before and after bevacizumab administration in both Groups LSP and ESP (p > 0.05 for all).

**Table 2:** Plaque features depicted during standard and contrast-enhanced ultrasonography.

	Group ESP	Group LSP	Controls	P value
	n = 18	n = 18	n = 19	
IMT, mm	0.24 ± 0.05*	0.27 ± 0.05^†^	0.45 ± 0.07	<0.001
Thickness, mm	0.84 ± 0.16	0.84 ± 0.08	0.80 ± 0.14	0.931
EI, dB	3.65 ± 1.43*^‡^	5.47 ± 1.48^†^	11.52 ± 2.62	<0.001
Ratio	0.33 ± 0.13*^‡^	0.48 ± 0.10^†^	0.67 ± 0.13	<0.001

Note: Data are expressed as the mean ± standard deviation. IMT = intimal-medial thickness; EI = enhanced intensity in the plaque; Ratio = the ratio of the enhanced intensity in the plaque to that in the lumen of the abdominal aorta.

^*†^p < 0.05 vs. controls.

^‡^p < 0.05 vs. Group LSP.

No significant difference in the maximum thickness of the plaques was observed among the three groups (p > 0.05). The control group exhibited a higher IMT, EI and Ratio compared with Groups ESP and LSP (p < 0.05). EI and Ratio in Group LSP were significantly increased compared with Group ESP (p < 0.05).

**Table 3:** Plaque morphological measurements by histological analysis.

	Group ESP	Group LSP	Controls	P value
	n = 18	n = 18	n = 19	
Plaque area, %	10.37 ± 2.99*	12.32 ± 2.53^†^	35.33 ± 2.05	<0.001
IMT, mm	0.22 ± 0.04*	0.20 ± 0.02^†^	0.42 ± 0.03	<0.001
Thickness, mm	0.79 ± 0.02*	0.80 ± 0.03^†^	0.85 ± 0.03	0.018

Note: Data are expressed as the mean ± standard deviation. IMT = intimal-medial thickness.

^*†^p < 0.05 vs. controls.

No significant difference in plaque area, IMT and the maximum thickness of the plaques was observed between Groups ESP and LSP (p > 0.05). The control group exhibited a higher IMT, maximum thickness of the plaques and a larger plaque area compared with Groups ESP and LSP (p < 0.05).
